# Machine translation and foreign language education

**DOI:** 10.3389/frai.2022.936111

**Published:** 2022-07-22

**Authors:** Per Urlaub, Eva Dessein

**Affiliations:** Global Languages, Massachusetts Institute of Technology, Cambridge, MA, United States

**Keywords:** education, second language acquisition, language instruction, machine translation, artificial intelligence, perceptions of technology, digital humanities, curriculum

## Abstract

Online machine translation tools have great potential to transform foreign language education. This essay will synthesize systematic research on the role of machine translation conducted in the field of educational linguistics. After describing approaches developed that promote the integration of machine translation into language learning environments, the essay will briefly outline lingering concerns associated with the integration of MT tools into educational settings. We will propose future R&D priorities that can generate products based on existing technologies that have the potential to support language learners more optimally compared to existing machine translation tools. We conclude that an acknowledgment of the difficulties of MT tools to handle socio-culturally complex source text would pave the way for the development of MT-based pedagogical tools.

## Introduction

The proliferation of impressively accurate consumer-oriented free online machine translation tools—in particular Google Translate—creates challenges and opportunities for language learners as well as the professional foreign language education community. After providing a general overview, this essay will highlight research in the field of educational linguistics that aims at analyzing both perceptions and the actual use of machine translation technologies among language learners and language instructors. This research review will focus on two interconnected clusters of research: (1) perceptions of MT tools among educators and language learners; (2) analysis and impact assessment of instructional approaches that use MT tool. Here, we will describe successful approaches developed by educational linguists that allow the integration of machine translation technologies into foreign language classrooms. After sketching these opportunities, we will outline what we consider the most significant risks associated with the integration of MT tools into language learning environments. We will argue that if deployed as an instructional technology, MT tools may lead to reductionist perceptions of language among students, teachers, and the general public. Based on these considerations, we will outline future priorities for both developers of machine translation applications and instructional designers that would generate modified technologies that support language learners in their quest to become both competent users of language technologies while developing autonomous proficiency in a foreign language with the help of innovative, pedagogically-enhanced MT tools.

## Literature review

Due to the limited scope of this essay, we will not be able to provide an exhaustive review of existing educational research on machine translation technologies in foreign language education. For a broad panorama of the existing research, we recommend Jolley and Maimone (2022) state-of-the-art article that meticulously reviews the existing research literature in the fields of educational linguistics, second language studies, and foreign language education. Their analysis of existing empirical research, systematic case studies, argumentative essays, and action research reports on MT in educational settings identifies five interconnected research clusters. We focus here only on two of these five clusters: (1) The first cluster consists of publications on MT use among language instructors and learners, as well as their perceptions, attitudes, and beliefs about the role of MT tools in formal language learning contexts. (2) The second cluster consists of publications that introduce and measure learning outcomes of in language classrooms that proactively and intentionally integrate MT technologies into the learning. We will describe these two clusters in the following two sections.

### How do educators and their students use and perceive machine translation applications?

In regards to learner and teacher perceptions, we know from systematic research that students unsurprisingly enjoy using machine translation apps while most teachers consider the use of machine translation apps unproductive, disruptive, and even often a form of academic dishonesty. In this context, we want to highlight a frequently-cited survey study conducted at Duke University among more than 900 undergraduate students. The data shows that learners frequently use online translators both in the context of their everyday life and in academic settings (Clifford et al., 2013). Many of the participating students report using Google Translate in a similar way to an online dictionary by entering individual words into the system. They also overwhelmingly report that they benefit from using online translators and that these tools should be allowed in their language courses (Clifford et al., 2013). This belief is surprising, considering that entering individual lexical items into online translators is an ineffective way to operate the technology. In order to tackle ambiguities, and accurately predict the meaning of synonyms, the technology relies heavily on the linguistic context that sentence-level entries offer. Contrary to student-users, instructors overwhelmingly do not recognize the pedagogical value of online translators in language classrooms, and many consider their use as a form of academic dishonesty (Clifford et al., 2013). We find similar concerns in visiting online discussion boards where language educators discuss pedagogical matters. Instructors fear that MT-tools reduce learner motivation and deprive them from opportunities to engage in critical cognitive processes that form the foundation of the language acquisition process.

These negative perceptions among teachers are consistent with a case study on teacher beliefs more recently analyzed by Hellmich (2019). Her data revealed a general skepticism of language instructors toward technology. As a result of these overall negative views, there are a number of studies that feature instructional units that aim at discouraging students from using online translators. For example, Steding (2009) and Faber and Turrero Garcia (2020) propose pedagogical suggestions that aim at highlighting the flaws of an online translator's linguistic output. These suggestions were developed for teachers to demonstrate to their students' why they prohibit the use of online translators.

Lastly, several studies conclude that an effective strategy for instructors who chose to prohibit the use of online translators and discourage students from violating this policy is to design tasks for homework assignments that are not compatible with the use of online translators (Ducar and Schocket, 2018; Henshaw, 2020). This strategy of designing what Ducar and Schocket coin a “Google-irrelevant classroom” is problematic, because tasks that are central to the development of L2 literacy, such as compositions, will either disappear or they will be administered during class time, thus eliminating valuable class time that otherwise would be dedicated to oral proficiency development.

### How do language educators integrate MT technologies successfully into learning environments?

In the early 2000s, educational linguists and instructional technologists started to investigate how to productively integrate online translators into their learning environment. Early on this work focused on instruction aimed at training professional translators and highlighted how using online translators in instructional settings could be effective (Somers, 2003). This is not surprising, as professional translators started to use translation software in the 1990s (Austermuehl, 2014). More recent articles on online translators in modern language instruction address more broadly the advantages and disadvantages of the implementation of online translators into educational settings (Niño, 2009; Benda, 2013; Ducar and Schocket, 2018; Valijärvi and Tarsoly, 2019).

Specifically, a subset of these studies focusses on instructional settings that focus on second language writing development as a particular promising environment for the integration of MT into conventional language instruction (Niño, 2008; Garcia and Pena, 2011; O'Neill, 2013, 2016, 2019; Correa, 2014; Kazemzadeh and Fard Kashani, 2014; Groves and Mundt, 2015; Stapleton and Kin, 2019; Tsai, 2019; Lee, 2020). Although most studies report increased motivation and improved L2 writing performance if students were trained to use online translators, the data is still insufficient to address the question of whether the use of online translators in instructional settings will prevent learners from developing the ability to produce written texts in the target language autonomously without the aid of online translators.

A number of educational linguists have studied how using online translators can result in elevating the metalinguistic awareness of language learners and empower learners by offering instant feedback on written and spoken language (Correa, 2014; Enkin and Mejias-Bikandi, 2016; Aikawa, 2018). The findings of these studies suggest that by using the technology to elicit feedback on both their written and spoken language, students can develop higher levels of linguistic awareness through the use of online translators.

## Discussion

### Risks associated with MT in language education

Today, there are no reasons for language educators and applied linguists to have any major concerns that relate to the semantic and morpho-syntactic accuracy of machine translators. We also believe that learning will not be substantially compromised in an environment where online translators are meaningfully integrated in the learning experience. There is however a true danger that the technology might lead to reductionist perceptions of language among students, teachers, and the general public. If proficiency becomes merely regarded as a tool, it reduces language as an exchange of messages in unsophisticated ways that do not recognize the sociocultural embeddedness of message and speaker. Such an instrumentalist notion of language proficiency does not resonate with the vast majority of educators and applied linguists, because it fails to acknowledge the richness and complexity of human interaction, identity, and culture. Instead, applied linguists and language educators understand proficiency as the ability to encode and decode meaning in a nuanced and context-sensitive way that is simultaneously a reflection and an expression of being and belonging. In addition to acknowledging the limitations of today's technology, educators must help learners at all levels interacting with machine translators to recognize these limitations. A language classroom that integrates machine translators must provide learners with experiences where they discover the limitations of machine translators.

### Recommendations for future developments

Freely available online translation applications do not only fulfill their primary role by serving users with impressively accurate translations, they also have the potential to support language learners to develop autonomous proficiency in a second language. However, in their current form, the user must uncritically accept the output as accurate without offering alternatives. Such static systems deny learners the opportunity to engage in a cognitive process that psycholinguists regard as central mechanism of the second language acquisition process: *Negotiation of meaning*. Through this process two or more interlocutors identify and resolve misunderstandings and communicative breakdown. These interactional patters are widely considered highly effective for language learner, because this repair-oriented process directs learners toward meaning-based as opposed toward grammar-based repair strategies (Ellis, 2003). In order to optimize MT tools for educational contexts, tools must engage with learners by providing them a meaningful interaction. Instead of providing a single translation that the leaners have to accept, the output must provide the learners with options that they can critically evaluate and thus engage in a process of negotiation of meaning through a human-machine collaboration. We therefore propose the following innovations.

#### Classic mode vs. educational mode

Online translation apps should offer two distinct modes of operation. In the “classic mode,” the machine translation platform operates in its conventional way. It offers users impressively accurate translations. In the “educational mode,” the platform provides users with opportunities to learn. Users get opportunities to critically integrate the system's output and as a result interact with the machine in order to collaboratively negotiate for meaning. As a result, learners will not only engage in cognitive processes that are essential for the acquisition of second language proficiency, they will also become more effective and critical users of MT tools.

#### Single translation vs. several version of translation

To optimize the users' language learning experience, the platform could provide a learner with three different possibilities to translate a phrase or a sentence. The learner can then choose one phrase and give a rationale for his/her decision. A (human) expert would then provide the student feedback both on their choice and their rationale. We believe that such a process can help learners to become more critical and thoughtful users of machine translation platforms, but it will also allow them to engage in some of the cognitive processes that support the second language acquisition process.

#### Feedback on the quality of the input

One of the major challenges (not only for learners, but also for conventional users of machine translation platforms) remains the ability to generate and modify input that is free of ambiguities and thus is less treacherous to handle for the machine translation platform. We believe that an “educational version” of a standard machine translation platform should also teach learners (and users) how to formulate effective input. For example, research on usage of MT applications among language learners shows that many use the technology to translate individual words. This approach is obviously an ineffective use of a system that relies on contextual information to predict meaning. Other users and learners enter unknowingly sentences that are highly ambiguous. In such cases, it would be beneficial if the translation platform would provide the user with feedback. For example, in response to a single word entry, the platform could ask for additional context, or it could provide several possible translations with explanations, so that the user can then choose the correct one. If the input is highly ambiguous, the platform could make the user aware of this fact and offer several less ambiguous suggestions that would teach the user eventually to operate the technology more effectively. It will also increase the learners' metalinguistic awareness both in their first language and in the target language.

## Conclusion

We believe that the above outlined innovations will accelerate the acceptability of machine translation technology in language education, because they would help transform a static technology into an interactive system that offers genuine opportunities to refine human-machine collaborations. More importantly, such more dynamic systems will offer the learners rich opportunities and convince language teachers to acknowledge the positive role that MT tools can play in their classrooms. We understand that while these innovations would only require minimal modifications at the level of the user interface, they may represent an implicit acknowledgment of the fallibility of MT technologies processing socio-culturally complex source texts. Whereas, such an implicit acknowledgment may not serve the interest of corporations that commercialize MT technologies and services, the educational community would benefit from a broader and more public acknowledgment of the imperfections of the technology, because it would open pathways to the development of MT-based pedagogical tools and thus help integrating MT technologies in sophisticated and productive ways into foreign language learning environments.

## Data availability statement

The original contributions presented in the study are included in the article/supplementary material, further inquiries can be directed to the corresponding author/s.

## Author contributions

All authors listed have made a substantial, direct, and intellectual contribution to the work and approved it for publication.

## Conflict of interest

The authors declare that the research was conducted in the absence of any commercial or financial relationships that could be construed as a potential conflict of interest.

## Publisher's note

All claims expressed in this article are solely those of the authors and do not necessarily represent those of their affiliated organizations, or those of the publisher, the editors and the reviewers. Any product that may be evaluated in this article, or claim that may be made by its manufacturer, is not guaranteed or endorsed by the publisher.
